# SOCRATES: an online tool leveraging a social contact data sharing initiative to assess mitigation strategies for COVID-19

**DOI:** 10.1186/s13104-020-05136-9

**Published:** 2020-06-16

**Authors:** Lander Willem, Thang Van Hoang, Sebastian Funk, Pietro Coletti, Philippe Beutels, Niel Hens

**Affiliations:** 1 Centre for Health Economic Research and Modelling Infectious Diseases, University of Antwerp, Antwerp, Belgium; 2 Interuniversity Institute of Biostatistics and Statistical Bioinformatics, Data Science Institute, Hasselt University, Hasselt, Belgium; 3 Centre for the Mathematical Modelling of Infectious Diseases, London School of Hygiene & Tropical Medicine, London, UK; 4 School of Public Health and Community Medicine, University of New South Wales, Sydney, Australia

**Keywords:** Social contact data, User interface, Transmission dynamics, Infectious diseases, Epidemics, Social distancing, Behavioral changes, Data sharing initiative, Open-source, COVID-19

## Abstract

**Objective:**

Establishing a social contact data sharing initiative and an interactive tool to assess mitigation strategies for COVID-19.

**Results:**

We organized data sharing of published social contact surveys via online repositories and formatting guidelines. We analyzed this social contact data in terms of weighted social contact matrices, next generation matrices, relative incidence and R$_{0}$. We incorporated location-specific physical distancing measures (e.g. school closure or at work) and capture their effect on transmission dynamics. All methods have been implemented in an online application based on R Shiny and applied to COVID-19 with age-specific susceptibility and infectiousness. Using our online tool with the available social contact data, we illustrate that physical distancing could have a considerable impact on reducing transmission for COVID-19. The effect itself depends on assumptions made about disease-specific characteristics and the choice of intervention(s).

## Introduction

Given the pandemic of SARS-CoV-2, which causes COVID-19 disease, it is of great importance to consider intervention strategies to slow down SARS-CoV-2 spread, and thus decrease surge capacity problems arising to health care provision and essential supplies [1, 2]. Physical distancing on a large scale, first at the epicenter of the outbreak in Wuhan, and later in other locations was shown to slow down SARS-CoV-2 spread (e.g. in Shanghai) [3].

Social contact surveys have proven to be an invaluable source of information about how people mix in the population [4–6] and explained close contact infectious disease data well [7–9]. For example, adapted social mixing during the A(H1N1)v2009 pandemic was fundamental to reproduce the observed incidence patterns [10]. In terms of prevention strategies, social contact data from the POLYMOD project [5] have been used to quantify the impact of school closure on the spread of airborne infections [11]. This was done by comparing the basic reproduction number R$_{0}$, or the average number of secondary infections caused by a single infectious individual in a completely susceptible population, derived from mixing patterns observed on weekends or during a holiday period with those derived from mixing patterns observed on weekdays.

In this research note, we highlight a social contact data sharing initiative and present an online tool to facilitate data access and analysis. Physical distancing measures can be mimicked with this tool by excluding the contribution of mixing patterns at specific locations to investigate the impact on disease transmission and guide policy makers. As a case study in light of COVID-19, we exploit our application to quantify the potential impact of school closure and physical distancing at work due to non-pharmaceutical interventions, a shift from common workplaces to teleworking at home or (temporary) unemployment.

## Main text

### Methods

Following a systematic literature review [4], corresponding authors were contacted to share their data subject to ethical approvals and GDPR compliance. All data have been refactored according to guidelines we developed during a Social Contact Data Hackaton in 2017 as part of the TransMID project. Each survey is split into multiple files to capture participant, contact, survey day, household and time-use data. For each data type, there is one “common” file and one “extra” file in which more specific variables related to the survey are included. Each data set contains a dictionary to interpret the columns (see http://www.socialcontactdata.org for more information).

To extrapolate survey data to the country level and obtain social contact rates on a weekly basis, we incorporate participant weights accounting for age and the number of observations during week (5/7) and weekend (2/7) days. We use the United Nation’s World Population Prospects [12] as reference and constrain weights to a maximum of 3 to limit the influence of single participants. The social contact matrix $m_{ij}$ can be estimated by:

1 $$\begin{aligned} m_{ij} = \frac{\sum _{t=1}^{T_i}w_{it}^d y_{ijt}}{\sum _{t=1}^{T_i}w_{it}^d}, \end{aligned}$$

where $w_{it}^{d}$ denotes the weight for participant *t* of age *i* who was surveyed on day type $d\in {\{\text{ weekday }, \text{ weekend }\}}$, $y_{ijt}$ denotes the reported number of contacts made by participant *t* of age *i* with someone of age *j* and $T_i$ denotes all participants of age *i*. By nature, contacts are reciprocal and thus $m_{ij}N_i$ should be equal to $m_{ji}N_j$. To resolve differences in reporting, reciprocity can be imposed by:

2 $$\begin{aligned} m_{ij}^\text {reciprocal} = \frac{m_{ij}N_i+m_{ji}N_j}{2N_i}, \end{aligned}$$

with $N_i$ and $N_j$ the population size in age class *i* and *j*, respectively [13]. This reciprocal behavior might not be valid for specific contact types, e.g. contacts at work for retail workers are most likely not contacts at work for their customers.

Transmission dynamics can be represented by the next generation matrix *G* with elements $g_{ij}$ that indicate the average number of secondary infections in age class *i* through the introduction of a single infectious individual of age class *j* into a fully susceptible population [14]. The next generation matrix is defined by:

3 $$\begin{aligned} G = DMq, \end{aligned}$$

with *D* the mean duration of infectiousness, *M* the contact matrix and *q* a proportionality factor [9, 11]. The proportionality factor *q* combines several disease-specific characteristics that are related to susceptibility and infectiousness. Equation 3 can be reformulated as:

4 $$\begin{aligned} g_{ij} = D * m_{ij} * s_i *k_j * \hat{q}, \end{aligned}$$

where $s_i$ denotes the susceptibility of age group *i*, $k_j$ the infectiousness of age group *j* and $\hat{q}$ other disease-specific factors. The leading right eigenvector of *G* is proportional to the expected incidence by age and R$_{0}$ can be calculated as the dominant eigenvalue of *G* [5].

To evaluate intervention strategies, we focus on the relative impact of adjusted social contact patterns on R$_{0}$ in line with the so-called *social contact hypothesis* [7] by cancelling disease specific features:

5 $$\begin{aligned} \frac{R_{0a}}{R_{0b}}&= \frac{\max (\text {eigen}(DM_aq)}{\max (\text {eigen} (DM_bq)} = \frac{\max (\text {eigen}(M_a*S*K))}{\max (\text {eigen}(M_b*S*K))}, \end{aligned}$$

where indices *a* and *b* refer to the different conditions, and S and K account for age-specific susceptibility and infectiousness, respectively [11]. Physical distancing can be evaluated by the elimination or reduction of location-specific subsets of the social contact data. Contacts reported at multiple locations are assigned to a single location in the following hierarchical order: home, work, school, transport, leisure and other locations. We simulate school closure by excluding all contacts reported at school. We evaluate physical distancing at work by applying a proportional reduction of the social contacts reported at work ($p_{\text {workplace}}^{\text {distancing}}$). To combine the effect of school closure and distancing at work, the social contact matrix *M* is calculated as:

6 $$\begin{aligned} M = M_\text {home} + (M_\text {work} * ( 1- p_\text {workplace}^\text {distancing})) + (M_\text {school} * 0) + M_\text {transport} + M_\text {leisure} + M_\text {other} \end{aligned}$$

We developed an interactive application to access and analyze social contact data based on R packages *shiny* [15] and *socialmixr* [16]. The user interface enables the selection of country-specific data, age categories, type of day, contact duration, intensity and gender. Using selection boxes, the user can opt to disable the assumption of reciprocity and participant weights. The user can also enable distancing strategies such as school closure or physical distancing at work, or include age-specific transmission parameters.

The user interface contains a plot of the social contact matrix and the principal results of the social contact analysis: *M*, relative incidences, the reference demography, participant statistics info on the data sets. Relative R$_{0}$ and *M* ratios are printed if reactive strategies are selected.

As COVID-19 case study, we estimate the effect of school closure and physical distancing at work on disease transmission dynamics. In order to do this, we use 3 age classes: 0–18 years, 19–60 years and over 60 years of age. For each country, we calculate contact rates after excluding data from holiday periods. We capture transmission dynamics with 0%, 20%, 40% and 60% distancing at work, with and without school closure. As proof of concept, we include the scenario where children are less vulnerable compared to elderly [$s_i = k_j = (0.5,1,1.5)$], instead of uniform susceptibility and infectiousness.

### Results

The http://www.socialcontactdata.org initiative, status 25th May 2020, includes data for Belgium, Finland, Germany, Italy, Luxembourg, Netherlands, Poland and the UK from POLYMOD [5], as well as data from other studies on social mixing in France [17], China [18], Hong Kong [19], Peru [20], UK [21], Russia [22], Zimbabwe [23], Vietnam [24], South Africa and Zambia [25]. All data are available on Zenodo [26–35] and can be retrieved within R using the *socialmixr* package.

**Fig. 1:**
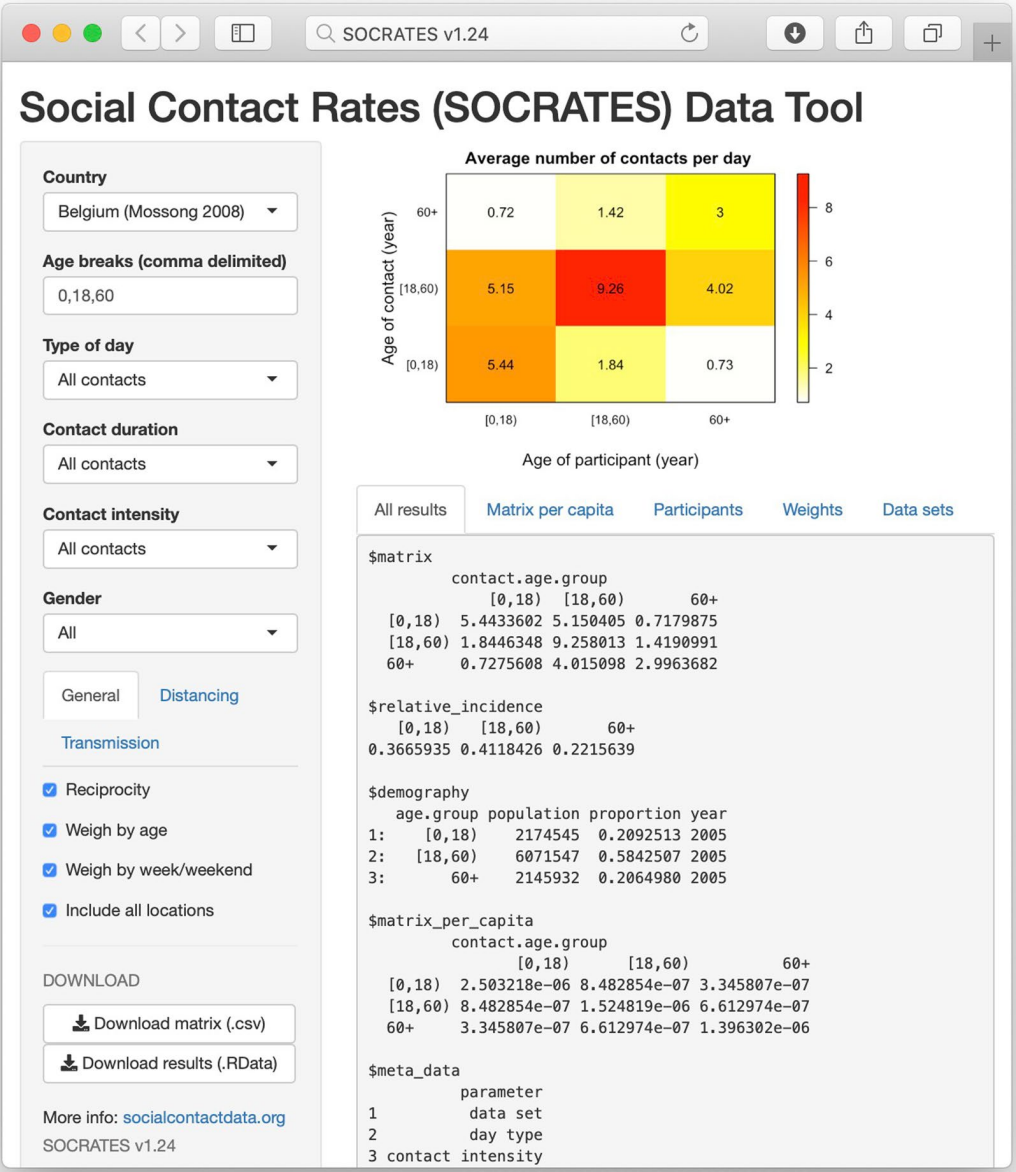
Screenshot of the online SOCRATES application [36]. The user interface enables the selection of country data in combination with temporal and contact features. The social contact matrix is shown on the right-hand side in addition to principal results and statistics. When users incorporate physical distancing at work or school closure, using the “Distancing” panel on the left-hand side, the R$_{0}$ ratio is added to the output (not shown)

The SOcial Contact RATES (Socrates) data tool [36, 37] enables quick and convenient generation of social contact matrices, relevant for the spread of infectious diseases. Figure 1 presents a screenshot of the user interface. The potential of using social contact patterns to simulate infectious disease transmission are endless, and we hope with this initiative to support data-driven modeling endeavors. The survey data from France and Zimbabwe contain multiple days per participant, hence we included only the first day for each participant to minimize the effect of reporting fatigue.

**Fig. 2:**
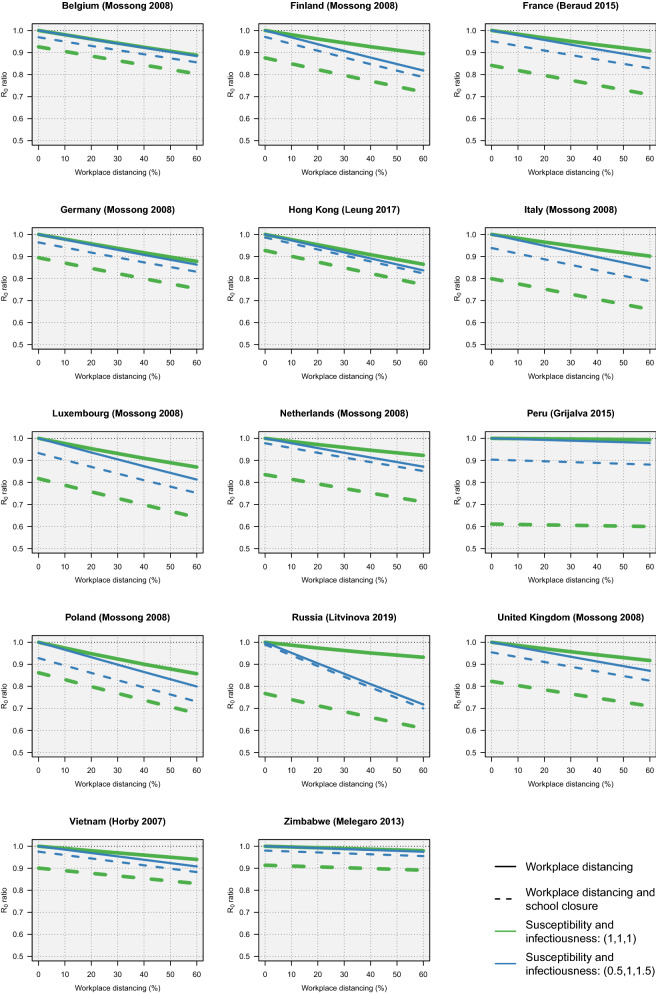
Predicted R$_{0}$ ratio by country due to physical distancing at work and/or school closure. The impact on R$_{0}$ is shown with uniform susceptible and infectiousness parameters (1,1,1) and when children are less vulnerable compared to elderly (0.5,1,1.5). The age classes are 0–18 years, 19–60 years and over 60 years of age. Distancing is incorporated by a reduction of location-specific social contacts

We demonstrate the effect of physical distancing at work and school closure on R$_{0}$ in Fig. 2. If we assume uniform susceptibility and infectiousness, we predict for most countries a 10% decrease in R$_{0}$ with workplace distancing of 60%. For Poland and Hong Kong, the reduction is slightly higher. The analysis for Peru shows little impact of workplace distancing since only few contacts were reported “at work”, whereas a substantial proportion of contacts was reported at the market or street. Cultural differences in how “at work” is understood should be considered when interpreting results. The data for Zimbabwe contains also relatively few reported contacts at work, which translates into a limited impact of workplace distancing in our analysis. The estimated R$_{0}$ reduction due to school closure is more country-specific, e.g. 10% reduction for Belgium and Vietnam, but 20% for Italy, Luxembourg and France. If we assume that elderly are more vulnerable compared to children, as might be the case for COVID-19 [38], the impact of school closure decreases dramatically. The positive effect of physical distancing at work on R$_{0}$ remains the same or increases.

**Fig. 3:**
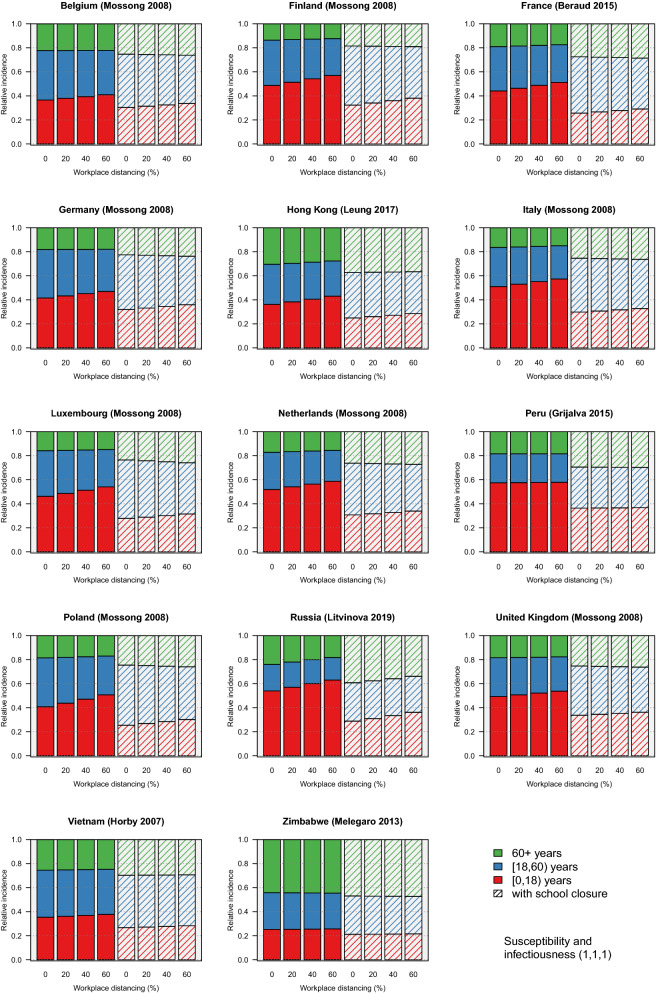
Predicted age-specific relative incidence by country with physical distancing at work and/or school closure. The analysis presented here does not account for age-specific vulnerability. Distancing is incorporated by a reduction of location-specific social contacts

The predicted relative incidences, as presented in Fig. 3, highlight the impact of school closure compared to an increase in physical distancing at work by age. The relative incidence in people 18–60 years of age decreases with an increasing proportion of workplace distancing, which is of interest if this age group is more vulnerable compared to children. The relative incidence in the age group above 60 years of age increases in all situations compared to no intervention. This does not imply that the absolute number of cases in this age group would rise.

## Limitations

Most survey designs were based on the POLYMOD survey though each survey had additional features and objectives which provide useful additional information. At the moment, we do not capture the full potential of each data set yet. Our case study elaborates on adapted school and work contacts and does not capture compensation behavior due to not being at school or work. This might be valid for a pandemic situation but not for regular (school) holidays. Social distancing due to (pandemic) scares are also not included yet.

The current application contains a local version of each data set, with some additional data reformatting. Our aim is to enable a direct link to Zenodo repositories. Note that some social contact surveys are available on Zenodo but not (yet) included in Socrates. E.g., the data from China [18] contains grouped contacts, which require different methodology. We omitted data from the UK [21], Zambia and South Africa [23] from our case study because only infants or adults were recruited.

Note that we will continue to develop this open-source tool [37] and thus the input/output/plots/scenarios might change in future editions.

## Data Availability

All data sets are available on Zenodo [26–34]. We also share all R-code on Zenodo [37].

## Abbreviation

Socrates: SOcial Contact RATES
